# Targeting Ataxia Telangiectasia-Mutated and Rad3-Related for Anaplastic Thyroid Cancer

**DOI:** 10.3390/cancers17030359

**Published:** 2025-01-22

**Authors:** Shu-Fu Lin, Chuen Hsueh, Wei-Yi Chen, Ting-Chao Chou, Richard J. Wong

**Affiliations:** 1 Division of Endocrinology and Metabolism, Department of Internal Medicine, New Taipei Municipal TuCheng Hospital, New Taipei City 23652, Taiwan; 2 College of Medicine, Chang Gung University, Taoyuan 33302, Taiwan; ch9211@cgmh.org.tw; 3 Department of Pathology, Chang Gung Memorial Hospital, Taoyuan 33305, Taiwan; 4 Institute of Biochemistry and Molecular Biology, National Yang Ming Chiao Tung University, Taipei 11221, Taiwan; chenwy@nycu.edu.tw; 5 Laboratory of Preclinical Pharmacology Core, Memorial Sloan Kettering Cancer Center, New York, NY 10065, USA; dtchou99@gmail.com; 6 Department of Surgery, Memorial Sloan Kettering Cancer Center, New York, NY 10065, USA; wongr@mskcc.org

**Keywords:** anaplastic thyroid cancer, ATR, BAY 1895344, kinase inhibitor, combination therapy

## Abstract

Background: Anaplastic thyroid cancer (ATC) is one of the most aggressive human malignancies and has a poor prognosis. Ataxia telangiectasia mutated and Rad3 related (ATR) is a key regulator for the DNA damage response and a potential target to treat cancer. Methods: We assessed the efficacy of BAY 1895344, an ATR inhibitor, in three ATC cell lines. Results: BAY 1895344 caused dose–response cytotoxicity in three ATC cell lines. BAY 1895344 induced S-phase and G2-phase arrest, activated caspase-3 activity and induced apoptosis in ATC cells. BAY 1895344 meaningfully retarded the tumor growth of an ATC xenograft model. BAY 1895344 therapy, combined with dabrafenib and trametinib, had synergism in vitro and revealed robust tumor growth suppression in vivo in two xenograft models of ATC harboring mutant *BRAF*^V600E^. Furthermore, the combination of BAY 1895344 with lenvatinib was more effective than either agent alone in a xenograft model of ATC. Conclusions: These results reveal that BAY 1895344 has potential in treating ATC.

## Simple Summary

BAY 1895344 is an Ataxia telangiectasia-mutated and Rad3-related (ATR) inhibitor that has shown activity against a variety of malignancies. We determined the efficacy of BAY 1895344 in the treatment of anaplastic thyroid cancer (ATC) in three ATC cell lines (8505C, 8305C, KAT18). BAY 1895344 induced cell cycle arrest in the S and G2 phases and caused apoptosis in these cell lines. BAY 1895344 monotherapy was able to repress the growth of the 8505C tumor model. BAY 1895344 enhanced the anti-tumor effect of lenvatinib in 8305C xenografts. In addition, BAY 1895344 increased the effects of dabrafenib and trametinib in treating 8505C and 8305C xenografts which harbor *BRAF^V^*^600E^, with acceptable safety profiles. Our results suggest the clinical efficacy of BAY 1895344 for ATC patient therapy.

## 1. Introduction

Anaplastic thyroid cancer (ATC) is a rare and aggressive disease with a dismal prognosis. The one-year survival rate is below 50% and the median overall survival is only 4–10 months with extensive multimodality therapy, including surgery, external beam radiotherapy, and chemotherapy [1,2,3,4]. In 2018, the US Food and Drug Administration (FDA) approved the combination of a BRAF inhibitor (dabrafenib) and a MEK inhibitor (trametinib) to treat ATC patients with *BRAF*^V600E^ mutation. However, only 10–50% of ATC patients harbor the *BRAF*^V600E^ mutation [5,6,7], and there are limited treatment options for ATC patients lacking this mutation. Furthermore, the median overall survival in ATC patients treated with dabrafenib and trametinib remains poor, at 10.4–14.5 months [8,9]. Adverse events associated with dabrafenib plus trametinib treatment that lead to treatment interruption occur frequently (17–50%) [9]. Novel therapies are needed for patients with ATC.

Cellular DNA is continually damaged due to exogenous and endogenous stressors [10]. DNA damage response (DDR) is a complex network of pathways that maintains genetic stability [10,11]. DDR leads to cell cycle arrest and DNA repair to ensure complete and accurate genome duplication [10,11,12]. Extensive unrepaired DNA damage can induce genomic instability, cell cycle arrest, senescence, and apoptosis [13,14,15]. Cancer cells usually lose at least one DDR pathway during malignant transformation [11,16]. This deficiency often diminishes the DDR capacity of cancer cells and leaves them more dependent on the remaining DDR pathways for DNA repair [17]. This characteristic offers opportunities for selectively killing cancer cells by targeting DDR pathways [18]. Some novel agents targeting DDR pathways have been developed to treat malignancies [18]. A study analyzed 126 samples of ATC, and the data revealed the most common genetic mutations were *TERT* promoter (75%), *TP53* (63%), *BRAF* (45%), *RAS* (22%) and *PIK3CA* (18%). *Ataxia telangiectasia-mutated (ATM)* and *DNA mismatch repair* alterations were identified in 7% and 8% of ATC, respectively [4].

The kinase Ataxia telangiectasia mutated and Rad3 related (ATR) is a key regulator of the DDR [12,19,20]. ATR is activated in response to a variety of DNA damage, including replication stress and single-stranded DNA [21]. The activation of ATR leads to the phosphorylation and activation of CHK1, which activates the G2/M cell cycle checkpoint, prevents unscheduled origin firing, maintains replication fork stability, and promotes DNA repair [22,23,24,25]. The inhibition of ATR induces DNA double-strand break (DBB) accumulation, an unstable genome and apoptosis [26]. Targeting ATR represents a promising strategy for treating cancers [26,27,28]. *ATR* is altered in 2.88% of multiple cancer types reported in an AACR Project GENIE Consortium study [29]. However, the prevalence of *ATR* alterations in ATC is unclear [4,30].

BAY 1895344 is a potent, highly selective and oral ATR inhibitor with a median-effect dose (IC_50_) in the nanomolar range [31]. BAY 1895344 has strong antiproliferative activity in a variety of cancer cell lines. BAY 1895344 monotherapy exhibits strong antitumor efficacy in xenograft models of ovarian, prostate, colorectal, breast cancer and mantle cell lymphoma. BAY 1895344 improves the antitumor activity of cisplatin for the treatment of ovarian cancer and olaparib for breast cancer [31]. BAY 1895344 has entered phase I clinical trial in patients with advanced solid tumors, including breast, colorectal, prostate, ovarian, endometrial, renal collecting duct, and appendiceal cancer. The most common treatment-emergent adverse events associated with BAY 1895344 treatment were hematologic toxicity, fatigue and nausea. Most adverse events were manageable and reversible [32]. We evaluated the effectiveness of BAY 1895344 in the treatment of ATC as single-drug therapy and combination therapy with multikinase inhibitors.

## 2. Materials and Methods

### 2.1. Cell Lines

Three human ATC cell lines (8505C, 8305C and KAT18) were evaluated in this study [33,34,35,36]. DNA short tandem repeat profiling was used for ATC cell line authentication and these cell lines were deposited in liquid nitrogen until use [36]. Then, 8505C and 8305C cells were cultured in MEM media supplemented with sodium bicarbonate (2.2 mg/mL) and sodium pyruvate (1 umol/mL). KAT18 cells were maintained in RPMI 1640 containing sodium bicarbonate (2.0 mg/mL). All media contained 10% fetal calf serum, 100 ug/mL streptomycin and 100 units/mL penicillin.

### 2.2. Drugs

BAY 1895344 and lenvatinib were purchased from MedChemExpress. Dabrafenib and trametinib were obtained from Novartis Pharma AG. For in vitro experiments, BAY 1895344, lenvatinib, dabrafenib and trametinib were dissolved in dimethyl sulfoxide (DMSO, Sigma) to a concentration of 10 mmol/L and stored at −80 °C until use. For in vivo studies, BAY 1895344 was diluted using 0.5% methyl cellulose (Sigma) as 12 mg/mL stock solution and stored at −80 °C. Lenvatinib was dissolved as12 mg/mL stock solution in hydroxypropyl methyl cellulose (Merck) and distilled water (1:200 *w*/*v*). Dabrafenib (8 mg/mL) and trametinib (0.16 mg/mL) were formulated in 0.5% hydroxypropyl methyl cellulose, 0.2% Tween 80 (Merck, Darmstadt, Germany) and distilled water.

### 2.3. Antibodies

Primary antibodies, including p-H2AX (Ser139), p-Histone H3 (Ser10), poly ADP-ribose polymerase (PARP), caspase-3 and proliferating cell nuclear antigen (PCNA), were obtained from Cell Signaling Technology. β-actin antibody and α-tubulin antibody were obtained from Sigma.

### 2.4. Cell Viability Assay and Synergy Analysis

Cells were seeded in 24-well plates in 1 mL of culture media at 2 × 10^3^ cells/well (8505C), 2 × 10^4^ cells/well (8305C) or 1 × 10^4^ cells/well (KAT18) and incubated overnight. A lowest cell density of 8505C cells was plated because 8505C cells had the fastest growth rate compared to KAT18 and 8305C cells, with the doubling time of 28.5, 39.4 and 41.6 h, respectively [37]. Cells were treated with six serial two-fold dilutions of BAY 1895344, starting from 1000 nmol/L, or placebo, and incubated for four days. Cell viability measurement was performed using the CytoTox 96 assay (Promega, Tokyo, Japan) to quantify lactate dehydrogenase (LDH) by spectrophotometry. Each condition was performed with 3 replicates. The IC_50_ of BAY 1895344 was determined after a 4-day treatment for each ATC cell line using the Chou-Talalay method and CompuSyn software (PD Science, Paramus, NJ, USA) [38,39].

Combination effects of BAY 1895344 and lenvatinib, or BAY 1895344 and dabrafenib plus trametinib, were analyzed. ATC cells were seeded as described above. ATC cells were treated with placebo, BAY 1895344, lenvatinib, dabrafenib plus trametinib or combination therapy for a four-day treatment period. Six sequential two-fold dilutions were assessed from the following starting doses for 8505C, 8305C and KAT18, respectively: BAY 1895344 at 306.8, 2709.2 and 495.2 nmol/; lenvatinib at 26.8, 116.8 and 102.0 μmol/L. Dabrafenib was assessed from 266.4 nmol/L and trametinib from 25.0 nmol/L for 8505C cells. For 8305C cells, dabrafenib was from 140.2 nmol/L and trametinib was from 28.0 nmol/L. These doses were chosen based on the IC_50_ of each drug in these cell lines determined in this and previous studies [40]. The combination index (CI) was determined using the Chou-Talalay method [38,39]. The resulting CI values provide quantitative data of antagonism (CI > 1), additive effect (CI = 1) and synergism (CI < 1) [38].

### 2.5. Western Blot Analysis

8505C, 8305C and KAT18 cells were seeded in 100 mm Petri dishes in 10 mL of media at 1 × 106 cells/dish overnight and incubated with BAY 1895344 (500 nmol/L) or control for 24 h and 48 h. Cell pellets were treated with lysis buffer (Cell Signaling Technology, Danvers, MA, USA), protease and phosphatase inhibitor mixture (Sigma, Burlington, VT, USA). The supernatant was collected after centrifugation. Protein (40 μg) was electrophoresed on 12% sodium dodecyl sulfate polyacrylamide gel electrophoresis gels, transferred onto polyvinylidene difluoride membranes, blocked with 5% skim milk in TBST at room temperature for 1 h, and exposed to rabbit p-H2AX (Ser139) primary antibody (1:1000) or rabbit PARP antibody (1:1000) at 4 °C overnight. Membranes were incubated with a secondary antibody conjugated to horseradish peroxidase. Band images were acquired using a digital imaging system (UVP ChemStudio touch PLUS, Analytik Jena, Jena, Germany) for signals developed by an enhanced chemiluminescence kit (PerkinElmer, Waltham, MA, USA).

### 2.6. Cell Cycle Analysis

The impact of BAY 1895344 treatment on cell cycle distribution was assessed using flow cytometry. ATC cells were seeded on 6-well plates at 4 × 10^5^ cells per well in 2 mL of media overnight. Vehicle or BAY 1895344 (500 nmol/L) was added and incubated for 48 h. Adherent cells were detached, collected, washed with phosphate-buffered saline (PBS) and fixed for 24 h with 70% ethanol at 4 °C. RNA was removed with Ribonuclease A (100 μg/mL, Sigma) and DNA was stained with propidium iodide (PI, 5 μg/mL; Sigma) for 15 min at 37 °C. DNA content was measured with flow cytometry (BD FACSCalibur Flow Cytometer, BD Biosciences, Franklin Lakes, NJ, USA). Each condition was performed in triplicate.

### 2.7. Apoptosis Assays

The impact of BAY 1895344 treatment on caspase-3 activity was measured using a fluorometric assay kit (Abcam, Cambridge, UK). ATC cells were plated on 100 mm Petri dishes at 2 × 10^5^ cells in 10 mL of media overnight. Vehicle or BAY 1895344 (500 nmol/L) was added and incubated for 48 h. Adherent cells were collected and lysed. Caspase-3 substrate (DEVD-AFC) and the reaction mixture were added and incubated for 1.5 h at 37 °C. Caspase-3 activity was measured using spectrophotometry (Infinite M200 PRO, Tecan, Männedorf, Switzerland). Each condition was performed in duplicate.

An Annexin V-Alexa Fluor 488 and PI kit (Thermo Fisher Scientific, Waltham, MA, USA) was used to determine the effects of BAY 1895344 treatment on early apoptosis. Cells were seeded in 6-well plates in 2 mL of media at 2 × 10^5^ cells/well overnight and incubated with BAY 1895344 (500 nmol/L) or vehicle for 48 h. Cells were then washed with PBS and incubated with Annexin V-Alexa Fluor 488 and PI at room temperature in the dark for 15 min. Stain cells were analyzed using flow cytometry (BD FACSCalibur Flow Cytometer, BD Biosciences). All experiments were performed in triplicate wells.

The efficacy of BAY 1895344 treatment leading to sub-G1 apoptosis was determined using flow cytometry. ATC cells were seeded in 6-well plates in 2 mL of media at 4 × 10^5^ cells/well overnight and treated with either vehicle or BAY 1895344 (500 nmol/L) for 48 h. Floating cells and trypsinized adherent cells were collected, and samples were prepared as described previously for cell cycle analyses. Sub-G1 apoptotic cells were measured using flow cytometry (BD FACSCali-bur Flow Cytometer, BD Biosciences). All experiments were performed in triplicate.

### 2.8. Immunofluorescence Microscopy

The impact of BAY 1895344 treatment on mitosis was assessed using immunofluorescence microscopy. ATC cells were seeded on 4-well chamber slides at 5 × 10^4^ cells in 1 mL of media overnight. Vehicle or BAY 1895344 (500 nmol/L) was added and incubated for 48 h. Cells were washed with PBS and fixed for 15 min using 4% paraformaldehyde (Sigma) at room temperature, washed with PBS, permeabilized for 10 min with 0.1% Triton X-100, washed with PBS and incubated with primary antibodies against rabbit p-Histone H3 (Ser10) (1:200) and mouse α-tubulin (1:1000) at 4 °C overnight. The cells were then washed with PBS and incubated for 25 min at 37 °C with secondary Alexa Fluor 488-conjugated goat anti-mouse antibody (1:1000, Thermo Fisher Scientific) and Alexa Fluor 633-conjugated goat anti-rabbit antibody (1:1000, Thermo Fisher Scientific). DNA was stained with 4′,6-diamidino-2-phenylindole (DAPI, 0.2 μg/mL; Thermo Fisher Scientific) for 10 min at room temperature. Mitotic cells were identified and the mitotic index was calculated for three ATC cell lines. Cells were imaged using a Leica TCS SP8 X confocal microscope (Leica Microsystems, Wetzlar, Germany).

### 2.9. Animals

8505C or 8305C cells (1 × 10^6^) were resuspended in 50 μL of culture medium, mixed with 50 μL of Matrigel at a 1:1 ratio and were subcutaneously injected into the right flanks of female athymic nude mice (7-week-old, National Laboratory Animal Center, Nangang, Taiwan). Tumor growth was measured with an electric caliper. Mice were randomized into six treatment groups, including placebo, BAY 1895344 (30 mg/kg, twice a day, 3 days on and 4 days off), lenvatinib (30 mg/kg, daily, 5 days on and 2 days off), dabrafenib plus trametinib (30 mg/kg and 0.6 mg/kg, respectively, daily, 5 days on and 2 days off), BAY 1895344 plus lenvatinib, or BAY 1895344 with dabrafenib plus trametinib by oral gavage. The 8505C and 8305C cells were chosen because they have high tumorigenesis rates. The doses of BAY 1895344, lenvatinib, dabrafenib and trametinib were chosen according previous reports [31,40]. The tumor size and body weight of each mouse were serially monitored twice a week. Tumor levels of p-H2AX (Ser139), cleaved caspase-3 and PCNA were assessed using Western blot analysis in mice with placebo or BAY 1895344 (30 mg/kg, twice a day) treatment. At indicated time points, carbon dioxide was applied for euthanasia. Tumors were excised, homogenized and sonicated on ice in protein extraction buffer (GE Healthcare, Chicago, IL, USA). Clarified supernatants were obtained after centrifugation and were aliquoted and stored at −80 °C until use. The Committee of Laboratory Animal Center at Chang Gung Memorial Hospital at Linkou approved the animal studies (No: 2021120604). All experiments were performed in accordance with animal protection and welfare regulations.

### 2.10. Statistical Analysis

Statistical analysis was performed using SPSS software version 26 (IBM). Results were expressed as mean ± standard error of the mean. Differences between two independent groups were analyzed using two-tailed, two-sample, Student’s t-tests with unequal variances. *p* < 0.05 was considered statistically significant.

## 3. Results

### 3.1. BAY 1895344 Induced Cytotoxic Effects in ATC Cell Lines

**Figure 1:**
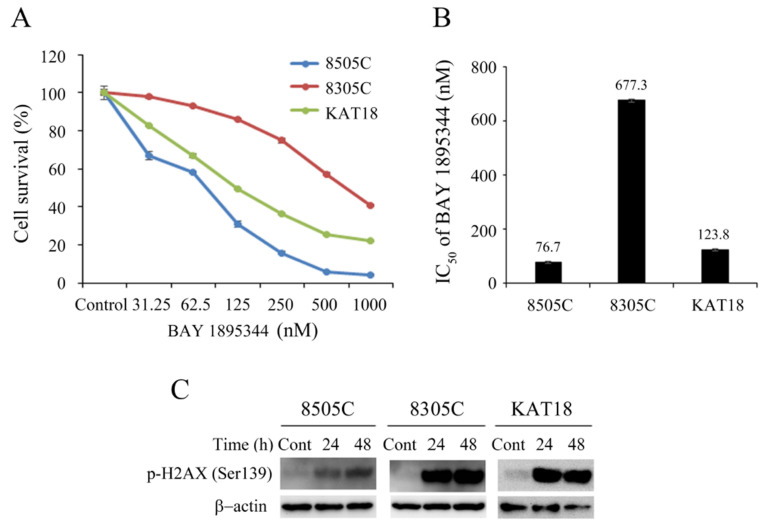
BAY 1895344 induced cytotoxicity and increased p-H2AX (Ser139) expression in three ATC cell lines. (**A**) Cytotoxicity was determined in ATC cells incubated with a series of six two-fold dilutions of BAY 1895344, beginning from 1000 nmol/L. Dose–response curves were acquired following a 4-day treatment in 8505C, 8305C and KAT 18 cell lines using LDH assays. (**B**) The median-effect dose (IC_50_) of BAY 1895344 on day 4 was obtained for three ATC cell lines using CompuSyn software. (**C**) The levels of p-H2AX (Ser139) were assessed with Western blot in 8505C, 8305C, and KAT 18 cells treated with placebo or BAY 1895344 (500 nmol/L) for 24 h and 48 h. BAY 1895344 increased p-H2AX expression by 24 h and the effect persisted until 48 h in three ATC cell lines. The uncropped blots are shown in Figure S1.

The cytotoxicity of BAY 1895344 was determined after a 4-day treatment in three ATC cell lines (Figure 1A). BAY 1895344 decreased cell survival in three ATC cell lines in a dose-dependent fashion. These cell lines showed diverse levels of susceptibility to BAY 1895344. BAY 1895344 at 1000 nmol/L inhibited cell growth by 95.6% (8505C), 59.3% (8305C) and 77.8% (KAT18) on day 4. The IC_50_ of BAY 1895344 in three ATC cell lines was assessed using CompuSyn software (Figure 1B). The 8505C cells had the lowest IC_50_ (76.7 ± 2.2 nmol/L) and the 8303C cells had the highest IC_50_ (677.3 ± 32.1 nmol/L). The IC_50_ doses of BAY 1895344 in these ATC cell lines were all below the clinically achievable serum concentration (2663.6 nmol/L) determined in a clinical trial [32].

### 3.2. BAY 1895344 Stimulated p-H2AX Expression

A prior report showed that BAY 1895344 increased the expression of p-H2AX, a marker of DNA DSBs, in lymphoma cell lines [31]. We evaluated the ability of BAY 1895344 (500 nmol/L) to increase p-H2AX expression in three ATC cell lines (Figure 1C). BAY 1895344 treatment significantly increased p-H2AX levels by 24 h and the effect persisted until 48 h in three ATC cell lines.

### 3.3. BAY 1895344 Promoted S-Phase and G2-Phase Cell Cycle Arrest

**Figure 2:**
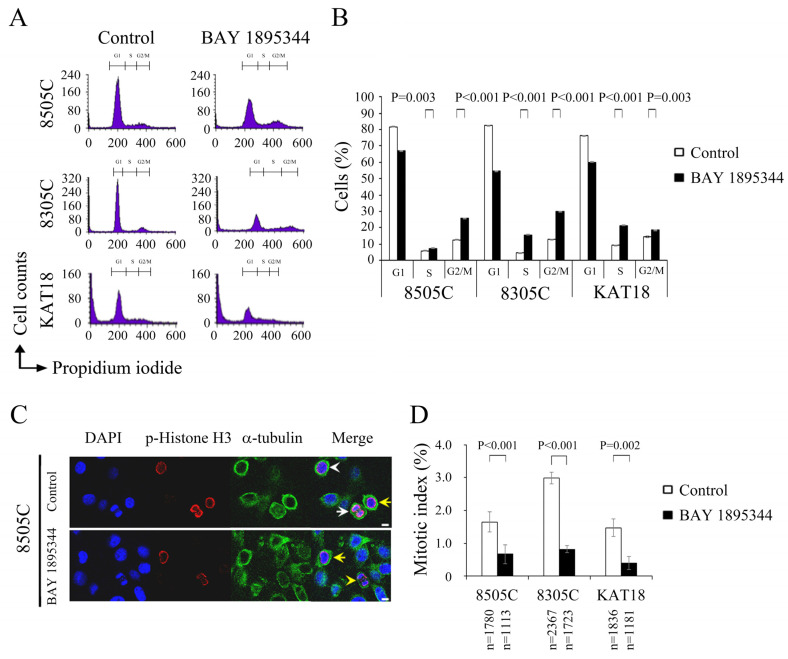
BAY 1895344 induced cell cycle arrest at the S phase and G2 phase. (**A**) Cell cycle analysis was performed using flow cytometry to measure DNA content in 8505C, 8305C and KAT 18 cells treated with placebo or BAY 1895344 (500 nmol/L) for 48 h. (**B**) BAY 1895344 treatment significantly increased the percentage of cells at the S and G2/M phase in 8505C, 8305C and KAT 18 cells. (**C**) Chromosomal appearance was evaluated in 8505C cells treated with BAY 1895344 (500 nmol/L) or placebo for 48 h using immunofluorescence confocal microscopy. Cells in the prophase (white arrowhead), prometaphase (yellow arrow), anaphase (white arrow), and telophase (yellow arrowhead) were identified. (**D**) The proportion of ATC cells in mitosis was assessed after treatment with placebo or BAY 1895344 (500 nmol/L) for 48 h. Cells were stained with DAPI and chromosome characteristics were assessed using immunofluorescence confocal microscopy. Mitotic index was assessed with a minimum of 1113 cells counted from at least ten different fields for each condition. BAY 1895344 significantly decreased the percentage of cells in mitosis in three ATC cell lines. Scale bar, 10 μm.

The effects of BAY 1895344 (500 nmol/L for 48 h) on cell cycle progression were evaluated in three ATC cell lines (Figure 2A). BAY 1895344 treatment significantly increased the percentage of cells in the S phase over that of the placebo treatment in 8505C (7.4 ± 0.3% and 5.9 ± 0.2%, *p* = 0.003), 8305C (15.6 ± 0.2% and 4.4 ± 0.1%, *p* < 0.001) and KAT18 (21.3 ± 0.5% and 9.3 ± 0.2%, *p* < 0.001), demonstrating BAY 1895344 induced S-phase arrest in three ATC cell lines. In addition to S-phase blocking, we found BAY 1895344 treatment significantly increased the proportion of cells in the G2/M phase than control treatment in 8505C (25.5 ± 0.2% and 12.4 ± 0.1%, *p* < 0.001), 8305C (29.9 ± 0.2% and 12.7 ± 0.0%, *p* < 0.001) and KAT18 (18.5 ± 0.7% and 14.4 ± 0.2%, *p* = 0.003) cells (Figure 2B), demonstrating that BAY 1895344 arrested cells in the G2/M phase.

Immunofluorescence confocal microscopy was applied to evaluate the effect of BAY 1895344 (500 nmol/L for 48 h) on mitosis. A representative cell line (8505C) is shown in Figure 2C. Mitotic cells were visualized, and the mitotic index was calculated with a minimum of 1113 cells counted from at least 10 different fields for each condition for three ATC cell lines. BAY 1895344 treatment substantially reduced the proportion of mitotic cells compared to the control in 8505C (0.7 ± 0.1% and 1.7 ± 0.2%, *p* < 0.001), 8305C (0.8 ± 0.2% and 3.0 ± 0.2%, *p* < 0.001) and KAT18 (0.4 ± 0.2% and 1.5 ± 0.2%, *p* = 0.002) cells (Figure 2D), demonstrating that BAY 1895344 decreased cells in mitosis.

### 3.4. BAY 1895344 Treatment Stimulated Apoptosis

**Figure 3:**
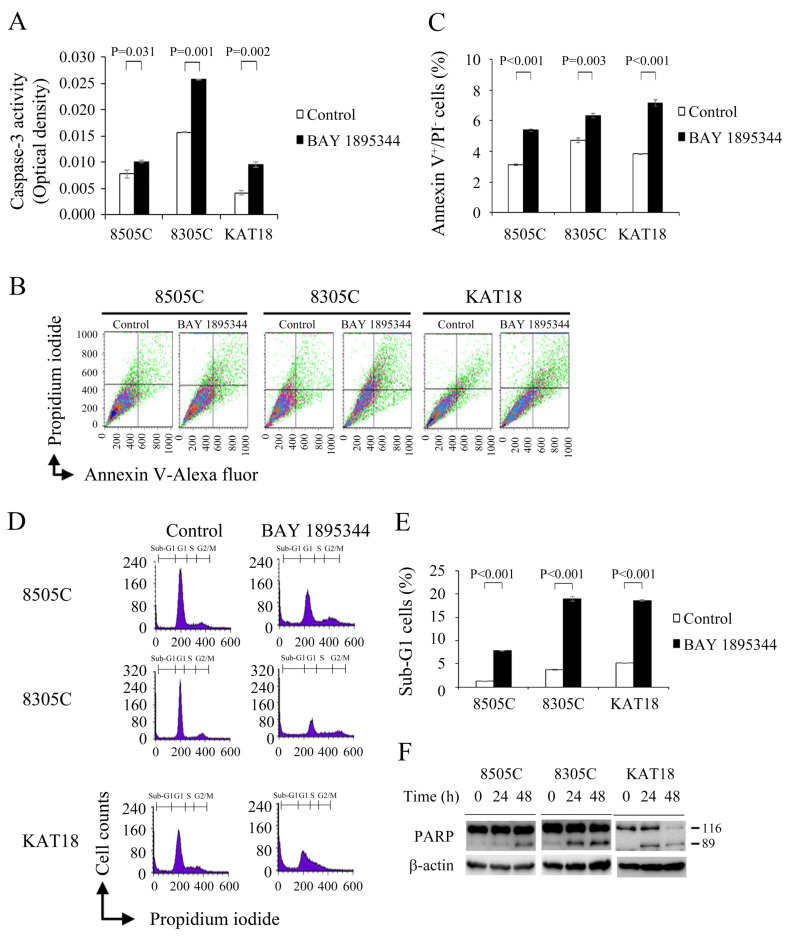
BAY 1895344 increased caspase-3 activity and induced apoptosis in ATC cells. (**A**) The change in caspase-3 activity was measured using a fluorometric assay kit in 8505C, 8305C and KAT18 cells treated with placebo or BAY 1895344 (500 nmol/L) for 48 h. BAY 1895344 significantly increased caspase-3 activity in three ATC cell lines. (**B**) 8505C, 8305C and KAT18 cells were incubated with BAY 1895344 (500 nmol/L) or placebo for 48 h and early apoptosis was determined by an Annexin V FITC kit to detect Annexin V-positive/PI-negative staining using flow cytometry. (**C**) The statistical analysis of three independent experiments for each condition described in (**B**) showed that the percentage of early apoptotic cells was significantly increased with BAY 1895344 treatment in three ATC cell lines. (**D**) Sub-G1 apoptotic cells were detected by assessing the cellular DNA content with fluorescent flow cytometry in 8505C, 8305C and KAT18 cells treated with vehicle or BAY 1895344 (500 nmol/L) for 48 h. (**E**) The statistical analysis from three independent experiments for each condition described in (**D**) revealed the proportion of sub-G1 apoptotic cells was significantly increased with BAY 1895344 treatment in three ATC cell lines. (**F**) ATC cells were treated with vehicle or BAY 1895344 (500 nmol/L) for 24 and 48 h. Immunoblotting analysis showed the levels of cleaved PARP, a marker of apoptosis, were markedly increased following BAY 1895344 treatment by 48 h in 8505C cells and by 24 h in 8305C and KAT18 cells. The uncropped blots are shown in Figure S1.

The elimination of cancer cells through the induction of apoptosis is a cancer therapeutic strategy [41]. The inhibition of ATR induces apoptosis in alveolar rhabdomyosarcoma [42]. We evaluated the effects of BAY 1895344 (500 nmol/L for 48 h) on caspase-3, an executioner caspase in apoptosis in 8505C, 8305C and KAT18 cells (Figure 3A). BAY 1895344 meaningfully increased caspase-3 activity higher than the control treatment in 8505C (0.010 ± 0.000-optical density [OD] and 0.008 ± 0.000-OD, *p* = 0.031), 8305C (0.026 ± 0.000-OD and 0.016 ± 0.000-OD, *p* = 0.001) and KAT18 (0.01 ± 0.000-OD and 0.004 ± 0.000-OD, *p* = 0.002) cells.

BAY 1895344-mediated caspase-3 stimulation may induce apoptosis. The Alexa Fluor 488 Annexin V/PI apoptosis kit was used to determine the ability of BAY 1895344 (500 nmol/L for 48 h) to induce early apoptosis in three ATC cell lines (Figure 3B). The results reveal BAY 1895344 treatment significantly increased the proportions of early apoptotic cells compared to the control in 8505C (5.4 ± 0.1% and 3.1 ± 0.0%, *p* < 0.001), 8305C (6.3 ± 0.2% and 4.7 ± 0.1%, *p* = 0.003) and KAT18 (7.2 ± 0.0% and 3.9 ± 0.1%, *p* < 0.001) cells (Figure 3C).

The ability of BAY 1895344 (500 nmol/L for 48 h) to induce sub-G1 apoptosis in three ATC cell lines was evaluated (Figure 3D). Statistical analysis shows BAY 1895344 substantially increased the proportions of sub-G1 cells compared to the vehicle in 8505C (7.9 ± 0.0% and 1.2 ± 0.1%, *p* < 0.001), 8305C (19.0 ± 0.8% and 3.7 ± 0.1%, *p* < 0.001) and KAT18 (18.6 ± 0.4% and 5.1 ± 0.2%, *p* < 0.001) cells (Figure 3E).

Immunoblot was used to assess the levels of cleaved PARP, a marker of apoptosis, in ATC cell lines treated with BAY 1895344 (500 nmol/L) for 24 h and 48 h (Figure 3F). BAY 1895344 induced the expression of cleaved PARP by 24 h (8305C and KAT18) and 48 h (8505C), indicating that BAY 1895344 induced apoptosis in three ATC cell lines.

### 3.5. Synergistic Activity of BAY 1895344 with Kinase Inhibitors in ATC Cells

**Figure 4:**
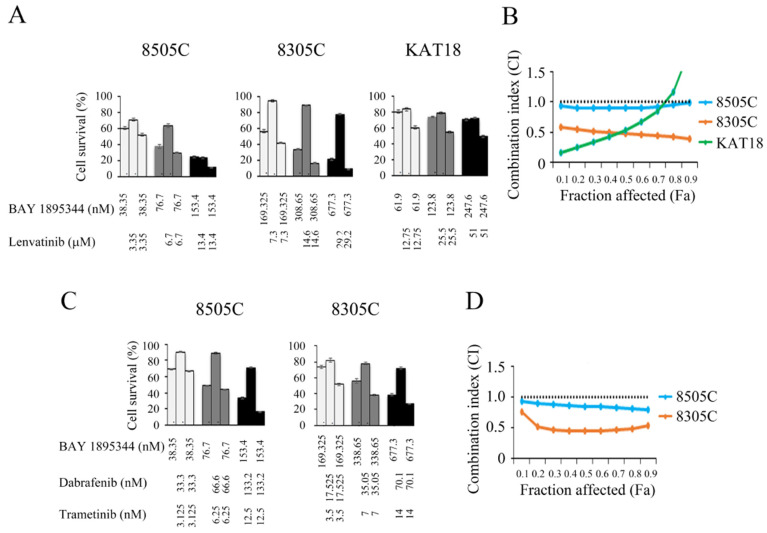
Combination therapies of BAY 1895344 with lenvatinib and BAY 1895344 with dabrafenib and trametinib in ATC cells. (**A**) The cytotoxicity of BAY 1895344, lenvatinib and combination treatment of BAY 1895344 and lenvatinib for a 4-day treatment was assessed in three ATC cell lines. The combination of BAY 1895344 and lenvatinib enhanced cytotoxic effects over single-agent treatment in three cell lines. (**B**) The combination index (CI) between BAY 1895344 and lenvatibib was calculated using CompuSyn software and plotted against the fraction affected (Fa) in 8505C, 8305C and KAT18 cells. BAY 1895344 plus lenvatinib had synergistic effects in 8305C, additive to synergistic in 8505C and synergistic to antagonistic in KAT18. (**C**) The cytotoxicity of BAY 1895344, dabrafenib plus trametinib and triple combination therapy following a 4-day incubation was evaluated in 8505C and 8305C cells. (**D**) CompuSyn software assessed the CI of BAY 1895344 and dabrafenib plus trametinib in 8505C and 8305C cells. The combination of BAY 1895344, dabrafenib and trametinib had synergistic effects in 8505C and 8305C cell lines. The horizontal dotted line represents CI = 1.

A phase 2 trial reveals lenvatinib was not an effective treatment for patients with ATC because the median progression-free survival was only 2.6 months and the median overall survival was 3.2 months. However, more than half of ATC patients experienced tumor shrinkage after lenvatinib treatment [43]. Future studies to evaluate lenvatinib in combination with other anticancer agents to increase the therapeutic effect of lenvatinib were suggested. We investigated the combination effect of BAY 1895344 and lenvatinib in three ATC cell lines (Figure 4A). The combination of BAY 1895344 and lenvatinib enhanced cytotoxicity more than either single agent alone. The CI was calculated to determine the interaction between BAY 1895344 and lenvatinib. The combination of BAY 1895344 and lenvatinib presented synergistic effects in 8305C. Synergism also appeared in 8505C when the fraction affected was < 0.9 and in KAT18 when the fraction affected was <0.8 (CI, 8505C = 0.9–1.0, 8305C = 0.4–0.59 and KAT18 = 0.16–1.86) (Figure 4B). The results reveal that the combination of BAY 1895344 and lenvatinib is mainly synergistic in three ATC cell lines.

The effect of BAY 1895344, dabrafenib and trametinib triple combination therapy in 8505C and 8305C cells that harbor *BRAF*^V600E^ was assessed [44]. Triple combination therapy increased cytotoxicity over single-modality therapy in these cell lines (Figure 4C). Interactions between BAY 1895344 and dabrafenib plus trametinib were evaluated (Figure 4D). This combination treatment had synergistic effects in both 8505C (CI, 0.79–0.93) and 8305C (CI, 0.53–0.75) cell lines.

### 3.6. Effects of BAY 1895344 and Kinase Inhibitors in ATC Xenograft Models

**Figure 5:**
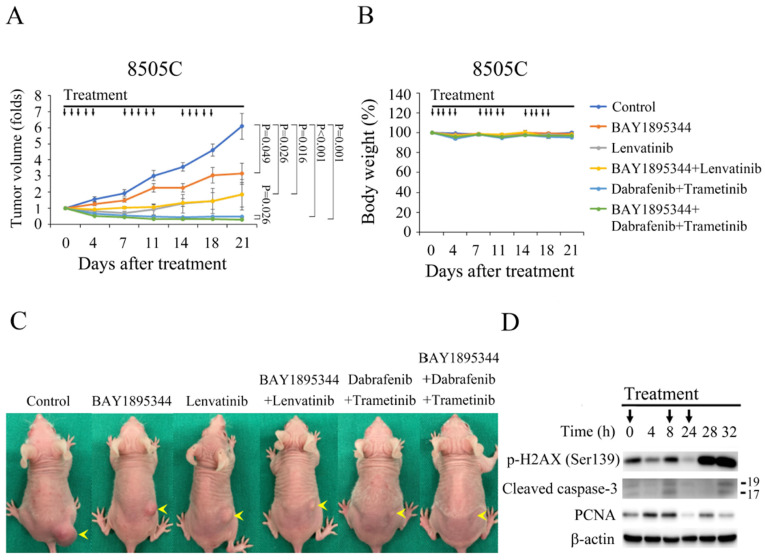
BAY 1895344 suppressed 8505C tumor growth and sensitized 8505C tumors to dabrafenib and trametinib therapy. (**A**) Mice with 8505C tumors were treated orally for three cycles of vehicle, BAY 1895344, lenvatinib, BAY 1895344 and lenvatinib, dabrafenib plus trametinib or the triple combination of BAY 1895344 with dabrafenib plus trametinib. BAY 1895344, lenvatinib, BAY 1895344 and lenvatinib, dabrafenib plus trametinib and the triple combination significantly reduced 8505C tumor growth when compared with the control treatment (*p* < 0.05 for all five comparisons). The combination of BAY 1895344 and lenvatinib did not significantly improve therapeutic efficacy compared to either single agent. Triple combination therapy of BAY 1895344 and dabrafenib plus trametinib therapy was superior in reducing 8505C tumor growth relative to either single regimen treatment. (**B**) Three weeks of treatment using dabrafenib plus trametinib was associated with a significant decrease in body weight as compared with the vehicle treatment, while the other treatment regimens did not lead to significant changes in body weight. (**C**) Representative images of mice with 8505C xenograft (arrowhead) were taken on day 21 after treatment. (**D**) Levels of p-H2AX (Ser139), cleaved caspase-3 and PCNA following BAY 1895344 treatment in 8505C tumors was assessed using Western blot. p-H2AX (Ser139) was increased by 28 h and the effect persisted until 32 h. Cleaved caspase-3 was increased at 8 h and 32 h. PCNA was transiently increased at 4 h and 8 h, but significantly decreased by 24 h. Black arrow: treatment with placebo, BAY 1895344, lenvatinib, dabrafenib plus trametinib and combination therapies. The uncropped blots are shown in Figure S1.

We tested the therapeutic effects of BAY 1895344 monotherapy and the combination of BAY 1895344 with kinase inhibitors in vivo. Nude mice were implanted with 8505C cells injected into the right flanks subcutaneously. Therapy began 28 days after cells injection when the tumors reached a mean diameter of 5.7 mm. The mice were randomized into six groups and treated with vehicle (*n* = 4), BAY 1895344 (*n* = 4), lenvatinib (*n* = 4), BAY 1895344 and lenvatinib (*n* = 4), dabrafenib plus trametinib (*n* = 5) and the triple combination of BAY 1895344, dabrafenib and trametinib (*n* = 5) (Figure 5A). Compared with the vehicle, BAY 1895344, lenvatinib, BAY 1895344 and lenvatinib combination, dabrafenib plus trametinib and the triple combination therapy led to significant decreases in tumor growth after a 3-week treatment course (6.1 ± 0.8-fold, 3.2 ± 0.6-fold, 1.8 ± 1.0-fold, 1.8 ± 0.8-fold, 0.5 ± 0.0-fold, and 0.3 ± 0.0-fold, respectively; *p* < 0.05 for all five comparisons). The combination of BAY 1895344 and lenvatinib did not increase therapeutic efficacy over BAY 1895344 (*p* = 0.302) or lenvatinib (*p* = 0.997). However, triple combination significantly improved tumor volume response over BAY 1895344 (*p* = 0.009) and dabrafenib plus trametinib (*p* = 0.026). Serial treatment of BAY 1895344, lenvatinib, BAY 1895344 and lenvatinib combination and triple combination therapy did not result in significant changes in body weight as compared with the control treatment. Dabrafenib and trametinib slightly but significantly reduced body weight when compared with control mice on day 21 (95.3 ± 0.6% and 100.2 ± 1.2%, *p* = 0.021) (Figure 5B) [45]. However, dabrafenib and trametinib treatment led to a 4.7% loss in body weight which was below the maximum tolerated dose (<10% body weight loss). Representative mice were photographed on day 21 (Figure 5C). The molecular effects of BAY 1895344 treatment in 8505C tumors were evaluated (Figure 5D). Immunoblotting of tumors excised at varying time points revealed that p-H2AX (Ser139) was increased by 28 h and the effect persisted until 32 h. The expression of cleaved caspase-3, an active form of caspase-3, was increased at 8 h and 32 h. PCNA level, a biomarker of cell proliferation, was increased at 4 h and 8 h. However, significant degradation of PCNA occurred by 24 h.

**Figure 6:**
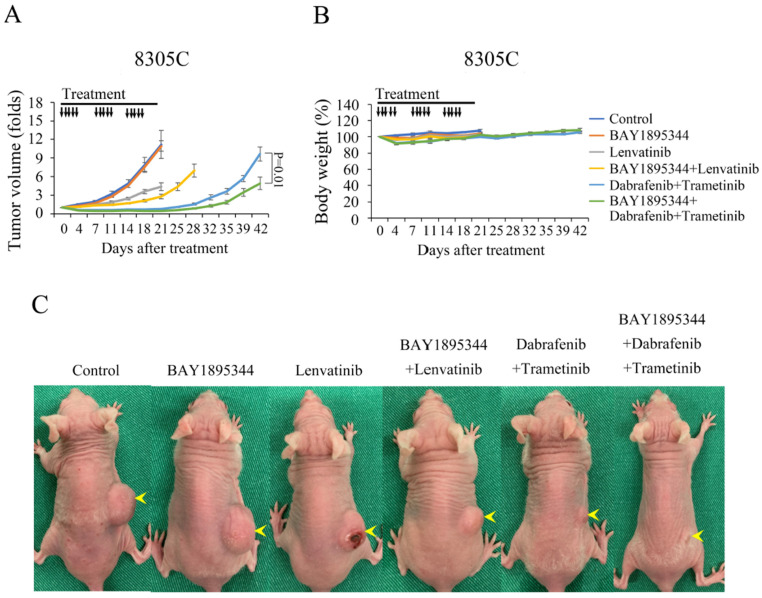
BAY 1895344 potentiated 8305C xenograft to lenvatinib and dabrafenib plus trametinib treatment. (**A**) Mice bearing 8305C flank tumors were treated orally with vehicle, BAY 1895344, lenvatinib, BAY 1895344 and lenvatinib, dabrafenib plus trametinib or the triple combination of BAY 1895344 with dabrafenib plus trametinib for 21 days. BAY 1895344 did not significantly inhibit 8305C tumor growth. Lenvatinib, BAY 1895344 and lenvatinib, dabrafenib plus trametinib and the triple combination therapy significantly retarded 8305C tumor growth compared to the control (*p* < 0.05 for all four comparisons). BAY 1895344 more significantly improved the therapeutic efficacy of lenvatinib and dabrafenib plus trametinib than either single-modality treatment during 21-day therapy. Tumor volume was persistently lower in the triple therapy group than the dabrafenib plus trametinib group on day 42. (**B**) BAY 1895344 did not significantly decrease body weight when compared with control mice after a 21-day treatment. Lenvatinib, BAY 1895344 and lenvatinib, dabrafenib plus trametinib and triple combination therapy slightly but meaningfully decreased body weight compared to control mice on day 21. (**C**) Representative photographs of mice with 8305C xenograft (arrowhead) were taken on day 21. Black arrow, treatment with placebo, BAY 1895344, lenvatinib, BAY 1895344 and lenvatinib, dabrafenib plus trametinib and the triple combination of BAY 1895344 and dabrafenib plus trametinib.

We further examined the therapeutic potential of BAY 1895344 monotherapy and in combination therapy in 8305C tumors. Mice with established 8305C xenografts with a mean diameter of 7.0 mm were randomized into six groups and treated with the vehicle (*n* = 7), BAY 1895344 (*n* = 7), lenvatinib (*n* = 6), BAY 1895344 and lenvatinib combination (*n* = 7), dabrafenib plus trametinib (*n* = 7) and the triple combination of BAY 1895344, dabrafenib and trametinib (*n* = 6) (Figure 6A). BAY 1895344 did not significantly retard tumor growth as compared to the control following a 21-day treatment (10.9 ± 0.8-fold and 11.2 ± 2.3-fold, *p* = 0.598). Lenvatinib, BAY 1895344 and lenvatinib combination, dabrafenib plus trametinib and the triple combination therapy significantly inhibited 8305C tumor growth compared to vehicle (4.4 ± 0.6-fold, 2.8 ± 0.4-fold, 0.8 ± 0.1-fold, 0.5 ± 0.0-fold and 11.2 ± 2.3-fold, respectively, *p* < 0.05 for all four comparisons). The combination of BAY 1895344 and lenvatinib repressed 8305C xenograft growth significantly more than BAY 1895344 or lenvatinib monotherapy (*p* = 0.007 and *p* = 0.037, respectively). In addition, triple combination increased tumor volume response more than BAY 1895344 or dabrafenib plus trametinib after a 21-day treatment course (*p* = 0.006 and *p* < 0.001, respectively). On day 42, when the treatment was discontinued for 21 days, tumor volume was still significantly lower in the triple therapy group than in the dabrafenib plus trametinib group (4.9 ± 1.0-fold and 9.7 ± 1.1-fold, *p* = 0.01), demonstrating the robust effects of triple combination therapy. Serial BAY 1895344 treatment did not induce significant body weight loss as compared with the control treatment on day 21 (103.6 ± 1.2% and 107.6 ± 1.5%, *p* = 0.055). However, lenvatinib, BAY 1895344 and lenvatinib, dabrafenib plus trametinib and triple combination therapy slightly but significantly decreased body weight when compared with control mice on day 21 (102.6 ± 1.3%, 100.4 ± 1.2%, 100.1 ± 1.2%, 102.8 ± 1.2%, and 107.6 ± 1.5%, *p* < 0.05 for all four comparisons) (Figure 6B). Representative photographs of 8305C tumors in mice were taken on day 21 (Figure 6C).

## 4. Discussion

We assessed the efficacy of an ATR inhibitor, BAY 1895344, in preclinical models of ATC. BAY 1895344 reduced cell proliferation in three ATC cell lines. BAY 1895344 repressed tumor growth in the 8505C tumor model, enhanced the therapeutic effect of dabrafenib and trametinib in 8505C and 8305C xenografts, and potentiated the antitumor effects of lenvatinib in 8305C tumors. These findings demonstrate the potential of targeting ATR using BAY 1895344 in ATC treatment.

Among three ATC cell lines evaluated in this study, 8505C cells had the lowest IC_50_ (76.7 nmol/L) and 8303C cells had the highest IC_50_ (677.3 nmol/L) of BAY 1895344. The most sensitive (8505C) and the most resistant (8305C) ATC cell lines determined from the in vitro study were evaluated in vivo. BAY 1895344 significantly inhibited tumor growth in the sensitive 8505C tumors, but not in resistant 8305C xenografts. These data are consistent with in vitro results. Though BAY 1895344 monotherapy was not sufficient to inhibit tumor growth in the 8305C model, BAY 1895344 potentiated the therapeutic effects of lenvatinib and dabrafenib plus trametinib in 8305C xenografts, which is clinically relevant since these drugs are used in patients with ATC.

BAY 1895344 therapy induced the expression of p-H2AX (Ser139), a marker of DNA DSBs, in three ATC cell lines. Our data are consistent with prior reports showing BAY 1895344 therapy has the ability to induce DNA DSBs in a variety of malignant cells [31,42]. DNA DSBs are one of the most deleterious types of DNA lesion that can lead to apoptosis [46].

The induction of apoptosis is an important strategy in cancer therapy because cancer cells frequently exhibit an ability to escape apoptosis by adopting strategies to over-ride apoptosis [41]. We found that BAY 1895344 increased caspase-3 activity and led to apoptotic cell death in three ATC cell lines, suggesting that apoptosis is one of the drug’s treatment mechanisms for ATC.

A previous report demonstrates that the inhibition of ATR led to S-phase cell cycle arrest in gastric cancer cells [47]. In consistence with this finding, BAY 1895344 accumulated cells in the S phase in three ATC cell lines. Besides S-phase cell cycle arrest, we noted BAY 1895344 blocked 8505C, 8305C and KAT18 cells in the G2 phase. In these ATC cell lines, BAY 1895344 increased the proportion of G2/M phase cells and reduced that of M-phase cells, indicating BAY 1895344 arrested cells in the G2 phase. Thus, S- and G2-phase block was likely another therapeutic mechanism of BAY 1895344 in ATC cells.

BAY 1895344 significantly inhibited 8505C xenograft tumor growth in this study. BAY 1895344 likely exhibits antitumoral effects in 8505C xenografts through apoptosis and cell cycle arrest, given that Western blot analysis showed increased cleaved caspase-3 expression and decreased PCNA level following BAY 1895344 treatment.

We explored the potential mechanisms of the increased cytotoxicity of combination treatment in 8505C cells. The combination of BAY 1895344 and lenvatinib and BAY1895344 and dabrafenib plus trametinib led to higher proportions of sub-G1 cells than either single-regimen therapy in 8505C cells (Supplementary Figure S2). These data indicate apoptosis was one of the underlying mechanisms of the improved cytotoxicity of combination therapies in 8505C cells.

The US FDA approved dabrafenib and trametinib combination for the treatment of ATC carrying *BRAF*^V600E^ mutation. However, refractory disease to this combination therapy develops in many patients [9]. Strategies to overcome drug resistance are needed. We found the robust antitumor activity of combined BAY 1895344, dabrafenib and trametinib against 8505C and 8305C xenografts in this study.

Our prior study has demonstrated that BAY 1895344 was effective in the treatment of differentiated thyroid cancer [48]. These encouraging results prompted us to evaluate the effects of BAY 1895344 for ATC. The current study additionally showed the efficacy of BAY 1895344 in treating ATC.

## 5. Conclusions

The ATR inhibitor, BAY 1895344, induced cytotoxicity in three ATC cell lines in vitro. In vivo analysis using 8505C and 8305C xenograft tumors demonstrated the therapeutic efficacy with acceptable safety profiles. Our results support the design of future clinical trials to evaluate the therapeutic effects of BAY 1895344 for patients with ATC.

## Data Availability

All data were contained within the article.

## Supplementary Materials

The following supporting information can be downloaded at https://www.mdpi.com/article/10.3390/cancers17030359/s1: Figure S1: Full pictures of the Western blots. Figure S2: The combination of BAY 1895344 and targeted therapies has higher potent effects to enhance apoptosis in 8505C cells. (A) The 8505C cells were treated with BAY 1895344 (153.4 nmol/L), lenvatinib (13.4 umol/L), a combination of BAY 1895344 and lenvatinib or placebo for 48 h and sub-G1 apoptotic cells were assessed. Combination of BAY 1895344 and lenvatinib led to higher proportions of sub-G1 cells than either single-drug therapy in 8505C cells. (B) The 8505C cells were treated with BAY 1895344 (153.4 nmol/L), dabrafenib (133.2 nmol/L) and trametinib (12.5 nmol/L), triple combination therapy of BAY 1895344, dabrafenib and trametinib or placebo for 48 h and sub-G1 apoptosis cells were assessed. Triple combination induced higher percentages of sub-G1 cells than either single-regimen therapy in 8505C cells.
