# Supplementary material for: Targeting Ataxia Telangiectasia-Mutated and Rad3-Related for Anaplastic Thyroid Cancer
Source: Cancers (Basel). 2025 Jan 22;17(3):359. doi: 10.3390/cancers17030359 (PMC11816221; doi:10.3390/cancers17030359)
Supplement: Supplementary file 1 [file cancers-17-00359-s001.zip › Figure S2.pdf]

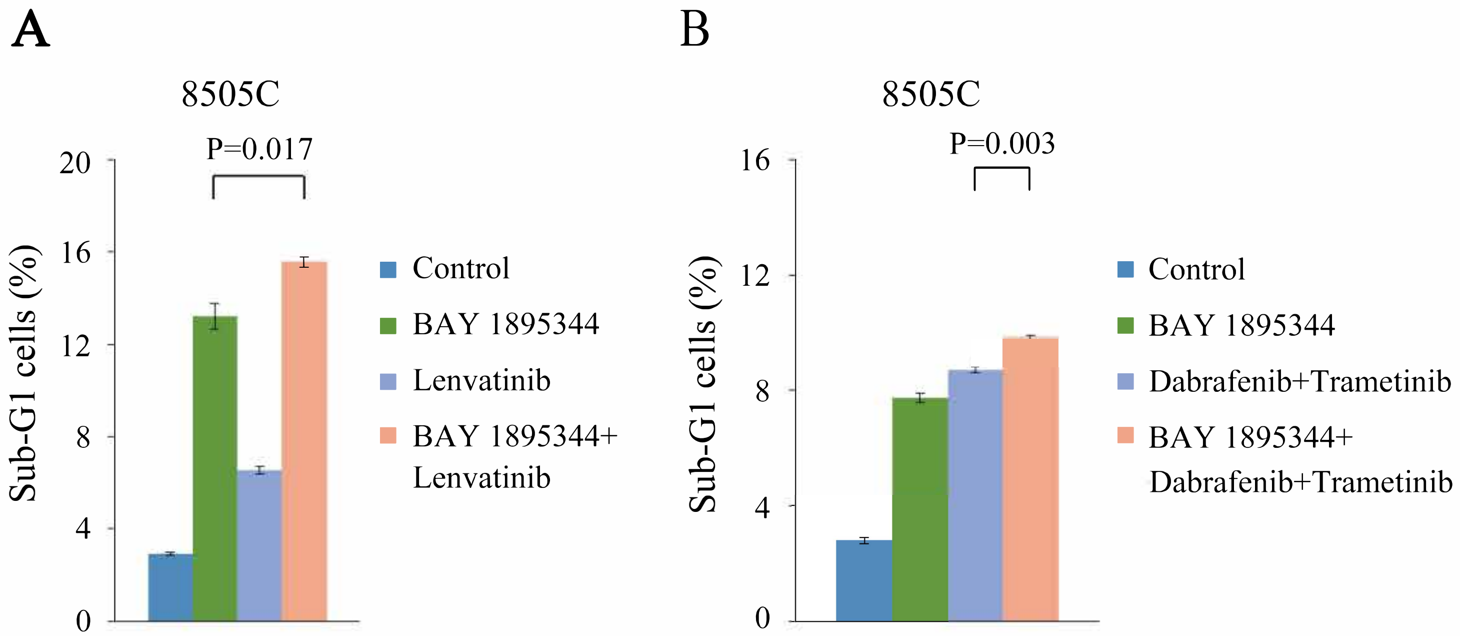

Figure S2. The combination of BAY 1895344 and targeted therapies has higher potent effects to enhance apoptosis in 8505C cells. (**A**) The 8505C cells were treated with BAY 1895344 (153.4 nmol/L), lenvatinib (13.4 umol/L), a combination of BAY 1895344 and lenvatinib or placebo for 48 h and sub-G1 apoptotic cells were assessed. Combination of BAY 1895344 and lenvatinib led to higher proportions of sub-G1 cells than either single-drug therapy in 8505C cells. (**B**) The 8505C cells were treated with BAY 1895344 (153.4 nmol/L), dabrafenib (133.2 nmol/L) and trametinib (12.5 nmol/L), triple combination therapy of BAY 1895344, dabrafenib and trametinib or placebo for 48 h and sub-G1 apoptosis cells were assessed. Triple combination induced higher percentages of sub-G1 cells than either single-regimen therapy in 8505C cells.
