# Supplementary figures and images for: Targeting Ataxia Telangiectasia-Mutated and Rad3-Related for Anaplastic Thyroid Cancer
Source: Cancers (Basel). 2025 Jan 22;17(3):359. doi: 10.3390/cancers17030359 (PMC11816221; doi:10.3390/cancers17030359)

Figure 1 (C)

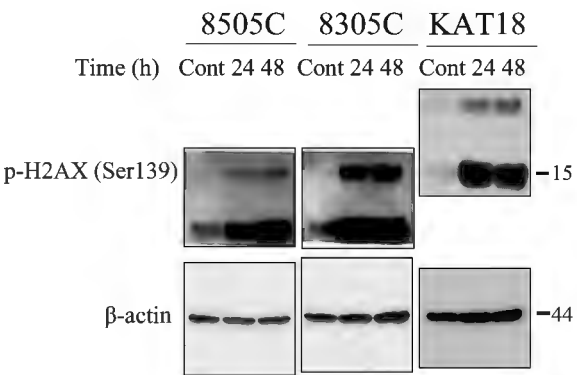

Figure 3 (F)

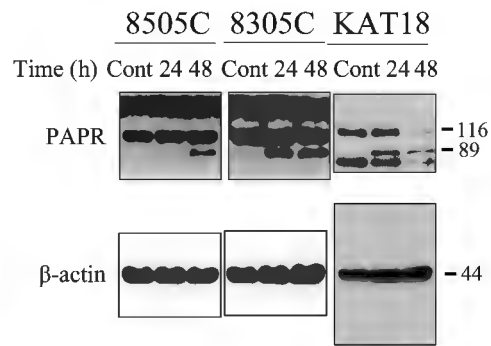

Figure 5 (D)

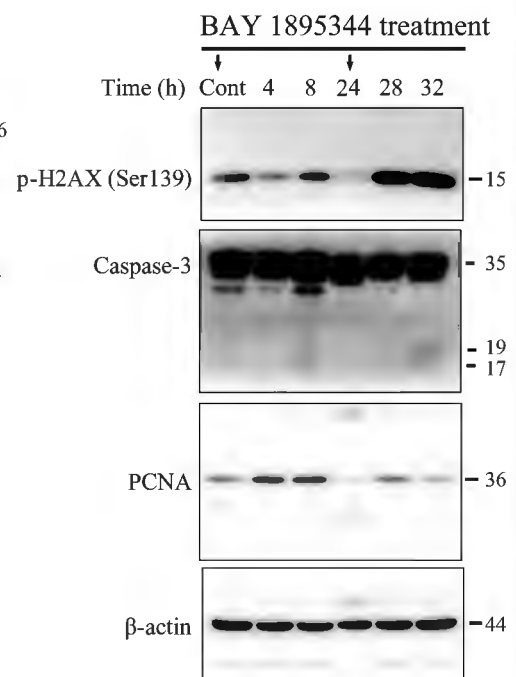

Supplement: Supplementary file 1 [file cancers-17-00359-s001.zip › Figure S1.pdf]
